# The Report of Dr. Monro and Sir Alexander Morison to the Lord Chancellor

**Published:** 1852-04-01

**Authors:** 


### *Official Reports, in the Case of Mrs. Cumming, to the Lord Chancellor and the Right Hon. the Lords Justices of the Court of Appeal.*

### THE REPORT OF DR. MONRO AND SIR ALEXANDER MORISON TO THE LORD CHANCELLOR.

"Cavendish-square, 11th Nov., 1851.

"My Lord—Having visited Mrs. Catherine Cumming for the purpose of examining the state of her mind, Edward Thomas Monro on the 6th and 8th inst., and Sir Alexander Morison on the 27th ult., we are of opinion that she is of unsound mind, upon the following grounds—viz., that she believes her daughters to be her enemies, and plotting against her; that she has every reason to believe that one of them attempted to strangle her, and that poison was given her in a cup, which, upon being analyzed by Dr. Barnes five years ago, was ascertained to contain oxalic acid, by which a fowl was afterwards killed.

"These impressions appear to engross her attention so entirely, that they must materially influence her every feeling towards them, and essentially affect the disposition of her property.

"We have the honour to remain,
"Your Lordship's most obedient servants,

"Edward Thomas Monro, M.D.
"Alexander Morison, M.D.

"To the Right Hon. the Lord Chancellor."

